# Improving Reproducibility in Synthetic Biology

**DOI:** 10.3389/fbioe.2019.00018

**Published:** 2019-02-11

**Authors:** Mathew M Jessop-Fabre, Nikolaus Sonnenschein

**Affiliations:** The Novo Nordisk Foundation Center for Biosustainability, Technical University of Denmark, Lyngby, Denmark

**Keywords:** synthetic biology, automation, reproducibility, cloud lab, biofoundry

## Abstract

Synthetic biology holds great promise to deliver transformative technologies to the world in the coming years. However, several challenges still remain to be addressed before it can deliver on its promises. One of the most important issues to address is the lack of reproducibility within research of the life sciences. This problem is beginning to be recognised by the community and solutions are being developed to tackle the problem. The recent emergence of automated facilities that are open for use by researchers (such as biofoundries and cloud labs) may be one of the ways that synthetic biologists can improve the quality and reproducibility of their work. In this perspective article, we outline these and some of the other technologies that are currently being developed which we believe may help to transform how synthetic biologists approach their research activities.

## Introduction

Science is reliant on the development of reproducible data. Despite the great importance of reproducibility, there have so far been few studies into the reproducibility of life science publications. One exception is within cancer biology, where it was reported that only 11% of landmark cancer studies could be reproduced (Begley and Ellis, 2012). In a survey of research scientists, 77% of biologists stated that they had tried and failed to reproduce someone else's result in the lab (Baker, 2016). And when asked “what percentage of published results in your field are reproducible?”, biologists estimated that only 59% of results are reproducible.

Different measures have been taken to maintain high levels of rigor within the sciences. The process of peer-review is one of these measures and has so far served as a successful strategy. However, with the increasing volume, complexity, and detail of data that is being published, peer-review is arguably unable to ensure that published research is reproducible and other measures are needed prior to this step.

**Figure 1:**
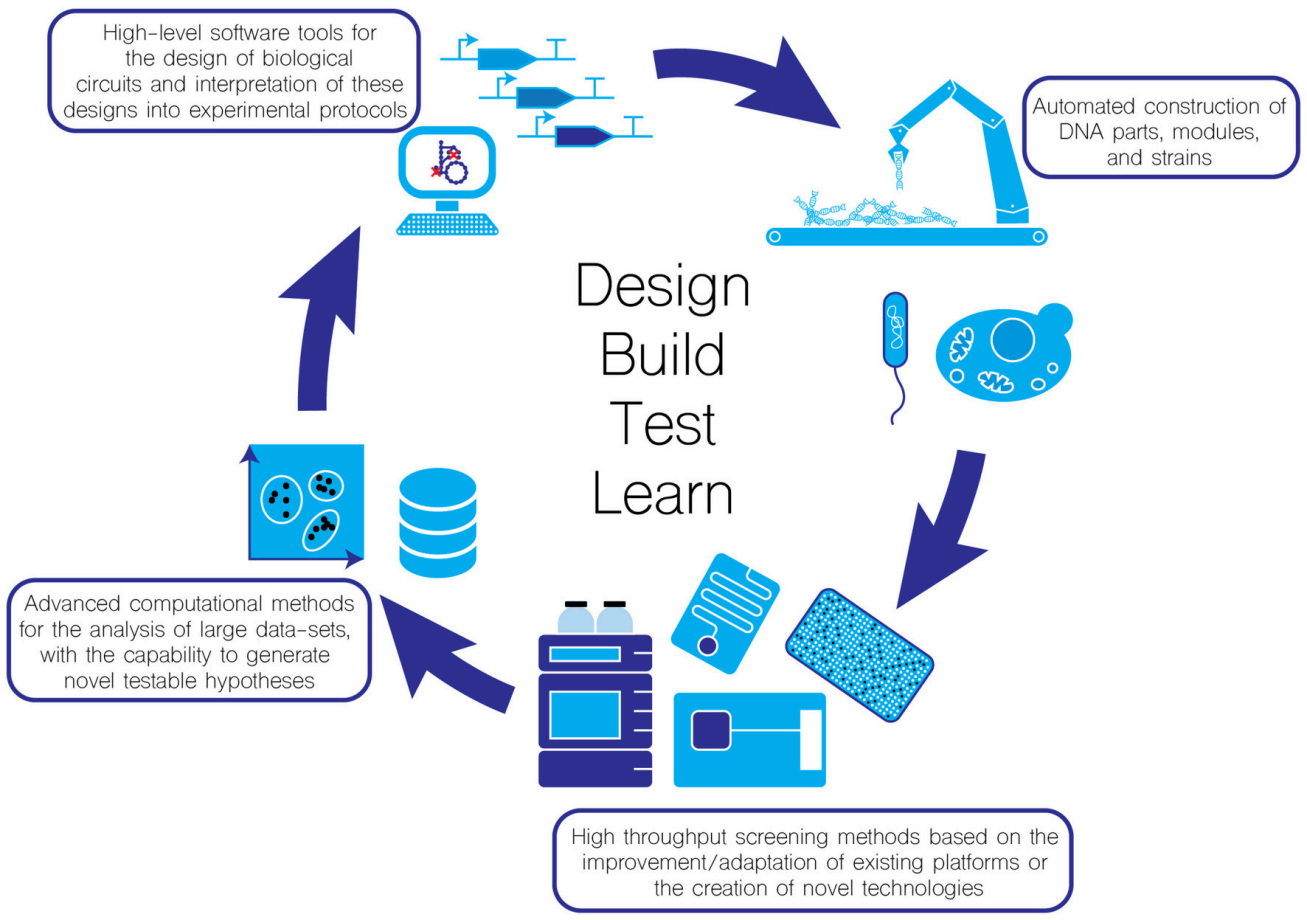
Moving towards automation in the Design-Test-Build-Learn cycle for enhancing the throughput and reliability of biological experimentation.

Synthetic biology aims to introduce engineering principles into the life sciences in the hope that it can greatly improve the reliability of the “Design-Build-Test-Learn cycle” (Figure 1). Any engineering discipline must be predictable and reproducible. In this perspective article, we discuss some of the ways the synthetic biology community is working towards achieving these goals.

## Improvement of Existing Laboratory Practices

One way to increase reproducibility is to increase the quality of protocol reporting. Experimental protocols are often specific to a laboratory or even to a single researcher, and are frequently taught to new lab members through a combination of practical guidance and a written protocol. This form of knowledge transfer is valuable in passing on tacit information (knowledge that is difficult to pass on in written form), but can lead to variation in the way experiments are performed. The passing on of written protocols without practical guidance can lead to even more problems, as written protocols are open to interpretation as they often contain ambiguities or rely on tacit knowledge (Miles and Lee, 2018). Online protocol editors, such as protocols.io, aim to improve the quality of protocols by creating a platform for scientists to easily share and edit protocol documents (Teytelman et al., 2016). Researchers are encouraged to upload detailed step-by-step instructions that can be followed, verified, and improved upon by researchers from other labs. In addition, videos can also be uploaded to capture some of the tacit information inherent in performing experiments. These protocols can then be linked to the methods sections of papers, and perhaps can help to improve the standardisation of common techniques and thereby improve experimental reproducibility.

Protocol editors combined with protocol management systems can provide a framework for labs to organise their research activities. The Aquarium software developed at the Klavins Lab is an example of such a system^1^. In this system a “designer” can accurately specify a protocol, which is then sent to the lab as a “job” to be performed by students, technicians, or robots. The system can be integrated with a laboratory information management system (LIMS) such as Benchling, enabling the use of materials to be tracked and giving trace-back of the items used for each experiment.

## Automation

For many years, laboratory automation has promised to revolutionise biology. However, the incorporation of traditional lab automation technologies (such as liquid handling robots) by academic labs has been slight. However, many of the techniques used within synthetic biology are performed on a routine basis, and as such may be desirable targets for automation. The successful implementation of automation technology can help to improve both the throughput and reproducibility of experiments, although the high cost and lack of flexibility of traditional lab automation has so far hindered their wide-spread adoption in academia. Recently though, there have been advances in automation technology that, when combined with new protocol sharing methods and protocol management systems, may allow for researchers to gain the benefits of automation.

The pharmaceutical industry has found particular favour with automation, as assay protocols are relatively simple to automate (Bogue, 2012). The high cash flow within large companies gives them the ability to invest in high quality and highly specialized equipment. This approach is unlikely to function well in an academic environment as the high costs and low flexibility of these operations are often prohibitive. Flexible and low cost solutions have generally not been offered by established vendors of liquid handling robots. Liquid handlers from vendors such as Tecan and Hamilton are state of the art, but expensive, large, and technically complex. In addition, operation of these machines usually requires skilled technicians, and can still take several months before they are operational. Interestingly, a recent Kickstarter campaign from Opentrons has aimed to address both the demand for a low cost and flexible liquid handler. The resulting OT-One robot has proved popular in both academia and industry, enabling Opentrons to establish itself in the field of lab automation. The more recent and advanced OT-2 liquid handler can be purchased from 4,000 USD, making it affordable to a wide range of academic labs. They have addressed the issue of flexibility through the adoption of open-source hardware and software. The relative ease of set-up and use of the Opentrons robots (programmable through a Python API, or soon through a graphical user interface) enables it to be used in a flexible manner by non-specialized workers. This means that many labs now have the opportunity to automate parts of their workflows and share their efforts throughout the community. The simple sharing of protocols will hopefully mean that other labs can easily test and verify the work of others.

Another issue that has been a problem for increased implementation of automation systems until recently, is that it has been difficult to link up devices from different manufacturers. Synthace is a start-up that aims to eliminate this issue by developing software that can control multiple robots from different vendors. An entire lab may be able to be controlled through a unified piece of software that allows for much simpler coordinated control of complex automation equipment.

Contrary to liquid handling robots, microfluidic technologies allow for the control of liquids on a microscopic scale and can also be used to automate and scale down many common laboratory procedures. Commercial microfluidic devices are becoming increasingly commonplace in the lab, with devices popular in the handling of DNA and RNA, such as fragment analysers used in next-generation sequencing workflows. The field of microfluidics is large and well-established, with over 3,000 articles published in 2017 alone (PubMed search for keyword: “microfluidic”). Microfluidics may provide a cheap and powerful alternative to traditional laboratory automation. Devices have been built that are capable of performing strain transformation and culturing, while other developments in the field have focused on DNA assembly (Ben Yehezkel et al., 2016; Gach et al., 2016; Linshiz et al., 2016). Even though microfluidic devices have been shown capable of automating protocols within synthetic biology, their adoption has been slow, and traditional liquid handlers remain the dominant method of automation, as the majority of laboratory automation has been designed to handle the array formats (such as 96 well plates) that are standard in the lab. Marrying microfluidic technologies to array-based laboratory automation can prove difficult, and is perhaps one reason for the limited integration of microfluidics into automated workflows.

Tedious and repetitive tasks are highly error-prone for humans to conduct, and automation may be seen as a way to reduce such error (Yeow et al., 2014). However, careful consideration for the requirements of any automation system must be carried out before the purchase of equipment. Counterintuitively, robots may be slower and less accurate than humans under certain conditions. Zielinski et al. (2014) reported that their newly developed pipetting protocol had up to a 3x larger coefficient of variation and took twice as long to perform when carried out by a robotic liquid handler rather than by a human. Traditional liquid handlers are also limited in ways such as requiring “dead volumes” of reagents inside wells, which can increase the cost of experiments. To maintain high levels of accuracy, all automation equipment must undergo strict and frequent quality assessments (Chai et al., 2013). There is an additional investment of time required for the set-up and optimisation of new protocols, which when combined with the aforementioned issues can dissuade academic labs from purchasing such systems. However, as we discuss below, there have recently been developments that aim to give researchers easier access to automation.

## Biofoundries

The issues mentioned in the previous section can prevent academic labs from investing in extensive automation. However, in the past few years there has been a movement to create fully or semi-automated labs termed “biofoundries.” Biofoundries typically consist of a core laboratory that has been extensively automated to carry out a range of functions. These integrated systems are unique in their flexibility compared to standard automated pipelines. A user can submit a job (e.g., create a combinatorial library) to the biofoundry, where it will be scheduled before being carried out by the automated lab. Once a job has been successfully run, the user is sent (if applicable) the biological output of their job along with any experimental or log data that the user requires.

Much of the work conducted within synthetic biology revolves around DNA manipulation, and as such so does the work of many of the biofoundries. One example is the Edinburgh Genome Foundry; whose focus is on manufacturing long lengths of genetic material *via* several DNA assembly methods. Their approach differs from that of a standard laboratory by only requiring a minimum of human intervention. A range of standard equipment, such as thermocyclers and incubators, are linked together by a network of robotic arms that can carry plates and samples between instruments. They are able to construct and transform complex sections of DNA into host organisms and then conduct a range of experiments on the engineered strains. The minimisation of human intervention, and shared use of reagent resources helps to bring down costs, hopefully to a point where it becomes economically viable for many researchers to outsource parts of their construction workflows to facilities like this.

The UK and US are currently leaders in the development of biofoundries, with several found throughout both countries (Chao et al., 2017). Many of these are open to accepting jobs from outside their host university institutes with the goal to serve the wider scientific community (Table 1). A widespread use of these facilities for design construction within synthetic biology may help improve standardisation within the field, if the community of biofoundries agree upon the design and adoption of operational standards.

**Table 1:** Overview of Biofoundries based at public institutions around the world.

**Name of biofoundry**	**Host organisation**	**Focus**	**Website**
Agile BioFoundry	US Department of Energy, University of California, USA	Full integration of the Design-Build-Test-Learn cycle, with advanced scale-up capabilities	https://agilebiofoundry.org
BIOFAB	University of Washington, USA	Use of the Aquarium software to control the functioning of a traditional lab. Services focus on plasmid construction and yeast strain construction.	http://www.uwbiofab.org
Concordia Genome Foundry	Concordia University, Canada	Cloning and NGS sample preparation	https://www.concordia.ca/research/casb/genome-foundry.html
DAMP lab	Boston University, USA	DNA construction, NGS preparations, protein expression and purification	https://www.damplab.org
Earlham DNA Foundry	Earlham Institute, UK	DNA assembly and insertion into a range of hosts, including plant cells	http://www.earlham.ac.uk/earlham-dna-foundry
Edinburgh Genome Foundry	Edinburgh University, UK	Assembly of large DNA fragments (>5,000 nt)	https://www.genomefoundry.org/home
GeneMill	University of Liverpool, UK	DNA construction, screening, and metabolomics workflows	https://genemill.liv.ac.uk
iBioFAB	University of Illinois, USA	Full automation of the Design-Build-Test-Learn cycle	https://www.igb.illinois.edu/research-areas/biosystems-design/research
London DNA Foundry	Imperial College, UK	Design and construction of complex DNA assemblies	http://www.londondnafoundry.co.uk
NUS Synthetic Biology Foundry	National University of Singapore, Singapore	DNA assembly, and analytical capabilities for downstream performance measurements	http://syncti.org/synthetic-biology-foundry/
SYNBIOCHEM	University of Manchester, UK	Automating the Design-Build-Test-Learn cycle for production of fine chemicals	http://synbiochem.co.uk/synbiochem-pipeline/

*This is not a comprehensive list, but contains those centres and facilities that have accessible webpages*.

## Cloud Laboratories

A similar concept to biofoundries is the “cloud laboratory.” Like a biofoundry, a cloud lab is usually comprised of a central facility that is heavily automated. However, the cloud lab gives a higher level of freedom to the customer by offering a real lab and its capabilities to biologists sat behind a computer in a remote location (Check Hayden, 2014). Two Californian companies have spearheaded this movement; Transcriptic, and the Emerald Cloud Lab. Both companies offer their extensive lab automation as a service, although each in a different way. Transcriptic has constructed a series of sterile work-cells. Within each work-cell is a set of basic lab equipment such as liquid handlers, thermocyclers, fridges, incubator, and a plate reader as well as a robotic arm to move materials between machines. Customers can control the actions within a work-cell *via* an application programming interface (API). A user can create an experimental protocol through the high-level Python API, which then converts the user's protocol into the JSON-formatted Autoprotocol language developed by Transcriptic to control their automation equipment (Miles and Lee, 2018). Users receive detailed results and diagnostics on their experiments, with troubleshooting on failed experiments made easier by the available metrics for all instruments on the executed run. The Emerald Cloud Lab has taken a different approach, where they have not broken down operations into discrete and identical work-cells, but have instead focused on offering a wider and more flexible range of services to the customer. The Emerald Cloud Lab has a stronger focus on the analytical side of biology through access to high-pressure liquid chromatography and mass spectrometry, among others. Cloud labs offer the chance for experimentalists to keep full control over their process, while also providing detailed and reproducible protocols. The hope is that academics will include these protocols within their publications, and share them online where others can use or modify them. Cloud labs may also enable early-stage biotech start-ups to test their early ideas and designs without the need for investing in setting up their own laboratory beforehand or entering into an incubator too early. However, there are still limitations with these technologies. Perhaps the most important is that it may be impossible to have any one facility that can cater to the needs of all biological researchers, since scientists make use of a wide array of instrumentation in their routine experiments. A recent review into cloud labs found that within biomedical research, 89% of published papers were found to contain one or more methods that could be carried out at a cloud lab (Groth and Cox, 2017). However, only 3% of papers had all of their methods supported by a cloud lab, suggesting that most complete workflows are as yet unable to be ported to a cloud lab. While it may not be necessary for a lab to implement complete workflows at a cloud lab, it may also prove disadvantageous to only perform a small part of their protocols (for example if it requires the frequent shipping of materials). There is therefore an inevitable judgement call that must be made to determine when it becomes beneficial to outsource a protocol.

## The Robot Scientist

The robust development of lab automation, whether in-house or through a biofoundry or cloud lab, may lead to biologists automating parts of their hypothesis generation and experimental design cycle too. While both biofoundries and cloud labs implement instructions from a human operator, the robot scientist aims to go one step further by removing the scientist from the hypothesis generation and experimental design steps. An early pioneering effort in this field was the creation of a “Robot Scientist” named Adam (King et al., 2004, 2009). The physical design of Adam resembles that of a biofoundry, with several instruments handling the execution of a set of experimental techniques. Materials are moved between these instruments by three robotic arms, and the entire set-up is enclosed in a sterile plastic enclosure. The main difference to a biofoundry is that Adam has generated its own scientific hypotheses, which it then tests experimentally, resulting in the generation of new scientific knowledge in yeast genetics. Adam is not alone. The more recent Eve Robot Scientist has also generated novel scientific insights, this time revealing new drug candidates for the treatment of *Plasmodium vivax* (Williams et al., 2015). This marriage of automation and basic hypothesis generation and testing may prove to be valuable within synthetic biology, as a researcher can be left to generate higher level hypotheses, which are then interpreted and executed by an automated facility.

## Standardisation

One of the key enablers of engineering disciplines is the adoption of a comprehensive set of standards (O'Connell, 1993). Such levels of standardisation are much more difficult to achieve in biological rather than electrical systems as there is a higher degree of uncertainty in the systems being engineered (Endy, 2005; Arkin, 2008; Canton et al., 2008). As an example, if one takes a regulatory element from one organism and places it in another organism then it is likely that the functionality of that element will differ. Even between different strains of the same species there is a large variation in how genetic elements are influenced by the host (Balagaddé et al., 2005; Cardinale et al., 2013). Synthetic biology requires standards that can cope with the issue of context dependency. Attempts to introduce standards, such as the biobrick standard, have been so far unable to fully deal with this issue (Decoene et al., 2018). However, promising work is being done to improve the orthogonality of biological parts (Stanton et al., 2013; Qian et al., 2017). Advances are also being made in to how biological parts are characterised and how orthogonality can be measured (Lucks et al., 2008; Mutalik et al., 2013; Ceroni et al., 2015). The creation of such standards may then help biofoundries and cloud labs to run standard building and characterisation protocols, that when combined with a developed set of operational standards can help to further increase the reliability and reproducibility of synthetic biology workflows. The standardisation of data practices is also just as vital, as the greatest of experimental results are worthless without the ability to learn from them, but such a discussion is out of the scope of this article [for a deeper discussion on data standardisation we refer the reader to Decoene et al. (2018)].

## Conclusions

One important question to address in synthetic biology is, how do we increase the predictability of our designed circuits and strains? Answering this question will have wide-reaching consequences for the field, but will require a shift in how synthetic biology is carried out in academia. Although synthetic biology has already broken the mould in many respects for how science is conducted at universities, deeper changes are needed in the funding and publishing infrastructures to guide the development of standardised practices.

In the meantime, recent developments in laboratory automation may help to improve the quality of experimental protocols, as machines must be programmed with precise and unambiguous instructions and protocols can be widely distributed and validated between labs around the world. The use of third-party facilities such as cloud labs or biofoundries can reduce some of the experimental variation between researchers while also reducing some of the burden. As we now order primers (and genes) instead of making them ourselves, perhaps we will soon too routinely order engineered strains. Robust lab automation holds the potential to bring computer-aided manufacturing (CAM) to synthetic biology by coupling computer-aided design (CAD) software for biological systems to the construction of cell factories, biological computers, and novel enzymes (Raman et al., 2009; Vasilev et al., 2011; Nielsen et al., 2016; Roehner et al., 2016; Cardoso et al., 2018).

There is an ever-growing demand for (high quality) data for machine learning applications, which requires a systematic approach to data generation, management, and sharing. Automation is capable of providing this approach as it can provide detailed logs of experimental runs and of the data acquisition from instruments. The data produced by academic labs needs to be made available to the greater scientific community in standardised formats, so that the greater community can learn from each other. Data collection and management is an area that requires a lot of attention. Although we have not had the space to offer a detailed discussion here, the synthetic biology community would greatly benefit from more focus on the standardisation of data practices. Traditional funding agencies are perhaps less likely to place emphasis on infrastructure, and so scientists are less incentivised to standardise their data practices.

## Author Contributions

MJ-F and NS conceived the study, and contributed jointly to the research and writing of this article.

### Conflict of Interest Statement

The authors declare that the research was conducted in the absence of any commercial or financial relationships that could be construed as a potential conflict of interest.

## Footnotes

^1^https://www.aquarium.bio
